# Supramolecular chemistry at the interface of biology, materials and medicine

**DOI:** 10.3762/bjoc.12.105

**Published:** 2016-05-31

**Authors:** Eric V Anslyn, Steven C Zimmerman

**Affiliations:** 1 Department of Chemistry, University of Texas, Austin, TX 78712, United States; 2 Department of Chemistry, University of Illinois at Urbana-Champaign, Urbana, Illinois 61801, United States

**Keywords:** biomimetic chemistry, host–guest chemistry, molecular recognition, molecular sensors

What do art, auto-mechanics, a rural Australian and Chinese village, two civil wars, and house building have to do with supramolecular chemistry? Unless you are an avid cover-to-cover reader of the Thematic Series of the *Beilstein Journal of Organic Chemistry* and happened upon the issue entitled “Supramolecular chemistry at the interface of biology, materials and medicine”, there is no possible way you would see the connection between these disparate items. In fact, these are part of the childhood recollections of several leading practitioners in the field of supramolecular chemistry and the authors of the mini-reviews in this Thematic Series. Before explaining the purpose of these recollections in more detail, an overview of this Thematic Series is needed.

We had two main goals in putting this Thematic Series together: (1) to highlight where the field of supramolecular chemistry is today, how it got there, and where it is going, and (2) to provide personal, autobiographies of leading practitioners of the field. The most important goal was to have a diverse group of experts in the field of supramolecular chemistry give an account of the state-of-the-art from their own unique perspective and subarea. There is an enormous amount still to learn about the fundamental nature of noncovalent interactions and particularly how to design and synthesize molecules that complex other molecules or are able to assemble spontaneously into three-dimensional structures. Thus, a major focus of the mini-reviews in this issue is on developing the supramolecular toolkit and better understanding how individual tools work and how they can be used to construct complex systems non-covalently.

The field of supramolecular chemistry has advanced over the past three decades and that success has allowed for a dramatic expansion in the field. In particular, much more attention is now paid to solving societal problems using supramolecular tools and the principles of supramolecular chemistry. The title of this Thematic Series was chosen to highlight three fields – biology, materials, and medicine – where the interface with chemistry has led to many important applications of supramolecular chemistry. Indeed, the reader will learn about how supramolecular discoveries in the laboratory have been translated into commercial products that can both improve and even save human lives.

It is now cliché to note that the earliest interest in supramolecular chemistry can be found in Emil Fischer’s famous lock and key analogy for enzymatic catalysis. If the origins of the field can be traced to that analogy made over a century ago, the event that propelled the field of supramolecular chemistry forward like no other occurred about thirty years ago. Thus, the joint 1987 Nobel Prize to Donald J. Cram, Jean-Marie Lehn, and Charles J. Pedersen “for their development and use of molecules with structure-specific interactions of high selectivity” [1] is often cited by senior members of the field as an inspirational event that both validated the field and signaled its future potential. The mini-reviews herein illustrate how that potential has been realized in multiple areas across a wide chemical landscape over the past thirty years. The articles also highlight future challenges and opportunities.

The second goal of this Thematic Series is more unusual. Over drinks at a workshop we wondered why our colleagues and we chose to pursue a career in supramolecular chemistry. Was it because as children we all played with Lego building blocks or Tinker Toys? Or was it some other reason? In turn, that got us thinking about the more human aspects of science. What did our parents do and how did they, along with our early life experiences influence our careers? What choices were made along the way and how did our research careers unfold? Rarely do students hear the stories behind the publications and the careers. Thus, the diversity in our authors was intended to have those stories reflect faculty at different stages of their careers, in different countries and with very different backgrounds.

At their core, these reviews are about the science of supramolecular chemistry and where the field is today and where it is going in the future. They also offer an intimate portrait of the people behind the work. We know that our authors greatly enjoyed telling their stories and we hope that the readers, particularly students, will find this perspective interesting and perhaps even helpful and inspirational.

Eric V. Anslyn and Steven C. Zimmerman

Austin, Urbana, May 2016
